# Neurovascular bundle preservation in robotic-assisted radical prostatectomy: How I do it after 15.000 cases

**DOI:** 10.1590/S1677-5538.IBJU.2022.99.04

**Published:** 2021-11-12

**Authors:** Marcio Covas Moschovas, Vipul Patel

**Affiliations:** 1 AdventHealth Global Robotics Institute Celebration FL USA AdventHealth Global Robotics Institute, Celebration, FL, USA

**Keywords:** Prostatic Neoplasms, Robotic Surgical Procedures, Prostatectomy

## Abstract

Despite the neuroanatomy knowledge of the prostate described initially in the 1980's and the robotic surgery advantages in terms of operative view magnification, potency outcomes following robotic-assisted radical prostatectomy still challenge surgeons and patients due to its multifactorial etiology. Recent studies performed in our center have described that, in addition to the surgical technique, some important factors are associated with erectile dysfunction (ED) following robotic-assisted radical prostatectomy (RARP). These include preoperative Sexual Health Inventory for Men (SHIM) score, age, preoperative Gleason score, and Charlson Comorbidity Index (CCI). After performing 15,000 cases, in this article we described our current Robotic-assisted Radical Prostatectomy technique with details and considerations regarding the optimal approach to neurovascular bundle preservation.

## INTRODUCTION

The surgical treatment for localized prostate cancer has been described beginning in the early 1900s (1). However, the lack of neuroanatomy knowledge associated with potency recovery following radical prostatectomy had led to high rates of erectile dysfunction at that time. Fortunately, in the 1980s, Walsh and Donker described the basis of prostate anatomy in their first report of nerve-sparing (NS) radical prostatectomy (2). This study marked the beginning of a new era by increasing postoperative potency rates and establishing the anatomic basis of erectile preservation in patients undergoing radical prostatectomy.

In the following years, open retropubic radical prostatectomy with nerve-sparing technique became the standard of care for patients diagnosed with localized prostate cancer. However, the advent of robotic surgery changed the standard treatment. But even with the advantages of this technology over the open and laparoscopic approaches, erectile outcomes remain a challenge for patients and surgeons (3–6). Recent studies have described that, in addition to the surgical technique, some important factors are associated with erectile dysfunction (ED) following robotic-assisted radical prostatectomy (RARP). These include preoperative Sexual Health Inventory for Men (SHIM) score, age, preoperative Gleason score, and Charlson Comorbidity Index (CCI) (7, 8). After performing 15,000 cases, in this article we describe our current RARP technique with details and considerations regarding the optimal approach to neurovascular preservation (9).

### Neurovascular bundles (NVB) anatomic considerations

Several authors have described the neuroanatomy and physiology of erectile function. These studies have shown that corpora cavernosa neurovascular supply preservation plays a crucial role in potency recovery following RP. By conserving the arterial supply of the pudendal artery and its variants (accessory pudenda), neural ischemia is minimized. In addition, preserving the cavernous nerves at the tip of the seminal vesicles also optimizes potency recovery (10, 11). Another critical factor for erectile preservation regards the intraoperative NVB neuropraxia by mechanical or thermal injury. Different classifications of neural injury have been described (12). In this scenario, extra care must be taken while manipulating the neural bundles on both sides of the prostate (13).

### Different degrees and planes of NVB preservation

We have previously described different anatomical studies regarding the grades of neurovascular bundle (NVB) preservation using the prostatic arteries as vasculature landmarks. The NVB preservation is based on the medial or lateral plane of dissection of these arteries. The Grades of dissection vary from Grade one (no nerve-sparing) to five (≥95% of nerve preservation) (14, 15). According to the authors, all patients from the study's cohort who were potent before surgery and underwent Grade 5 NS presented erections after surgery.

### Intrafascial

Intrafascial dissection represents the plane between the prostatic capsule and prostatic fascia at the posterolateral and anterolateral portions of the prostate. At this plane of dissection, the surgeon maximizes the NVB preservation achieving the best potency outcomes. However, this plane of dissection is associated with the highest positive surgical margins (PSM) rates in T3 tumors (16).

### Interfascial

The interfascial space is located between the prostatic fascia layers. The NVB preservation and postoperative erectile recovery of this dissection are inferior when compared to the Intrafascial approach. When accessing this plane, the lateral prostatic fascia is resected and is visualized attached to the final specimen (17).

### Extrafascial

This plane of dissection is located lateral to the prostatic fascia and is associated with complete NVB removal and the worst postoperative potency recovery. However, in terms of oncologic dissection, it is the safest and most indicated in patients with extracapsular extension (ECE) due to the increased margin removal (18, 19).

### Nerve-sparing RARP technique

Several authors have described different techniques to optimize the NVB preservation since Binder and Kramer described the first NS-RARP. In their study, ten patients with prostate cancer were operated on, and according to the authors, the NS technique combined the Walsh retrograde dissection with Campbell's anterograde approach (20). After more than 20 years since this first report, both approaches (anterograde and retrograde) are still used by current robotic surgeons.

### Anterograde NVB dissection

With this approach, the prostate is lifted by the seminal vesicles, and the NVB dissection is performed from the base to the apex. After creating the inferior plane between the Denonvilliers layers, the anterior dissection creates a space between the Denonvilliers fascia, lateral pelvic fascia, and prostate. Then, the prostatic pedicle is controlled with hem-o-lok clips or bipolar (8). In sequence, the NVB dissection is performed until the prostate apex.

### Veil of Aphrodite

Initially described by Manon et al. in 2006, this approach is also known as high anterior release of the prostate. A plane between the prostate capsule and fascia is created posteriorly at the base of the seminal vesicles. In sequence, the bilateral NVB release (posterolateral) is performed from 5 to 1 o’clock on the right side and from 7 to 11 o’clock on the left side. At the end of this dissection, the periprostatic tissue (Veil of Aphrodite) is suspended bilaterally, resembling a curtain from the pubourethral ligament (21, 22).

### Retrograde NVB dissection

In our routine, the retrograde release of the Neurovascular Bundles is the technique performed in all NS-RARP, despite the robotic approach (da Vinci Xi or da Vinci SP) (3, 4, 6, 8, 9, 23–27). After lifting the prostate by the seminal vesicles (SVs), we create a space between the Denonvilliers (DNV) layers. Then, we toggle the 30 degrees’ scope facing the posterior portion of prostate to perform the bilateral dissection from 5 to 1 o’clock on the right side and from 7 to 11 o’clock on the left side.

After releasing the posterior portion of the prostate, the dissection is performed from the apex to the base by incising the endopelvic fascia close to the prostate and communicating the lateral with the posterior planes. At this moment, the identification of the prostatic arteries, as mentioned before, guides the Degrees of NVB preservation. In some cases, it is not possible to visualize the posteromedial and anteromedial arteries. Therefore, we perform the dissection at the usual arterial topography and cautiously peel the NVB to reach the correct planes. Finally, the prostatic pedicles are controlled with hem-o-lok clips and athermal technique.

The postoperative potency outcomes of this technique were previously described by our group in a study comparing the anterograde with the retrograde approach. In this report, we have defined two groups of 172 patients who underwent NS-RARP, and the retrograde NVB dissection was associated with early potency recovery at 3, 6, and 9 months’ post-surgery (28).

Recently, we have described the modification of our technique by performing a modified apical dissection underneath the puboprostatic ligaments preserving the maximum amount of urethra length and periurethral tissues. This technique also preserves the lateral prostatic fascia in selected patients with small tumor burden (9). By adopting these modifications, we described improvements in the early potency and continence rates when comparing this approach with our previous technique.

### Our technical considerations for NS-RARP after 15.000 cases

Despite the surgical technique, the best series describing potency recovery following radical prostatectomy have never achieved 100% success rates. In this scenario, we have described several technical modifications to improve and maximize functional recovery over the years^7^. In our routine, the NVB preservation is planned according to the preoperative tumoral staging with imaging (MRI) and biopsy report (29). Knowing the tumor location, stage, and anatomical relation with the NVB is crucial for planning the Grades of dissection and preoperative counseling regarding the possible rates of potency recovery following surgery (15, 26).

**Figure 1:**
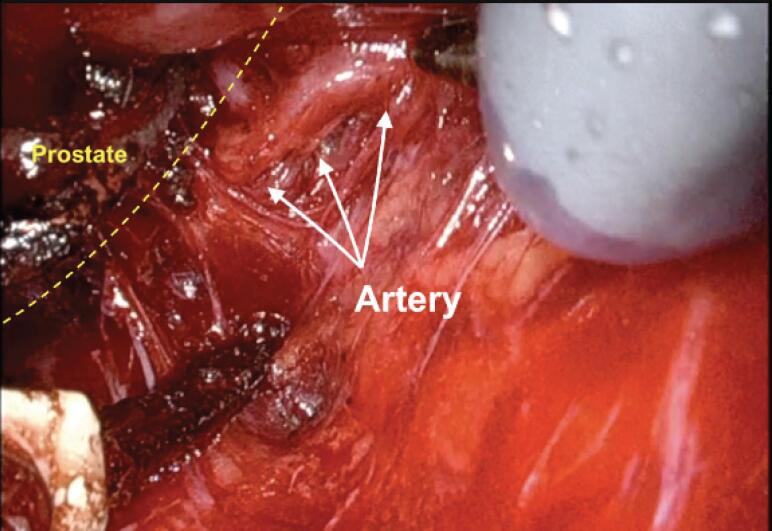
Posterior medial prostatic artery on the right side.

**Figure 2:**
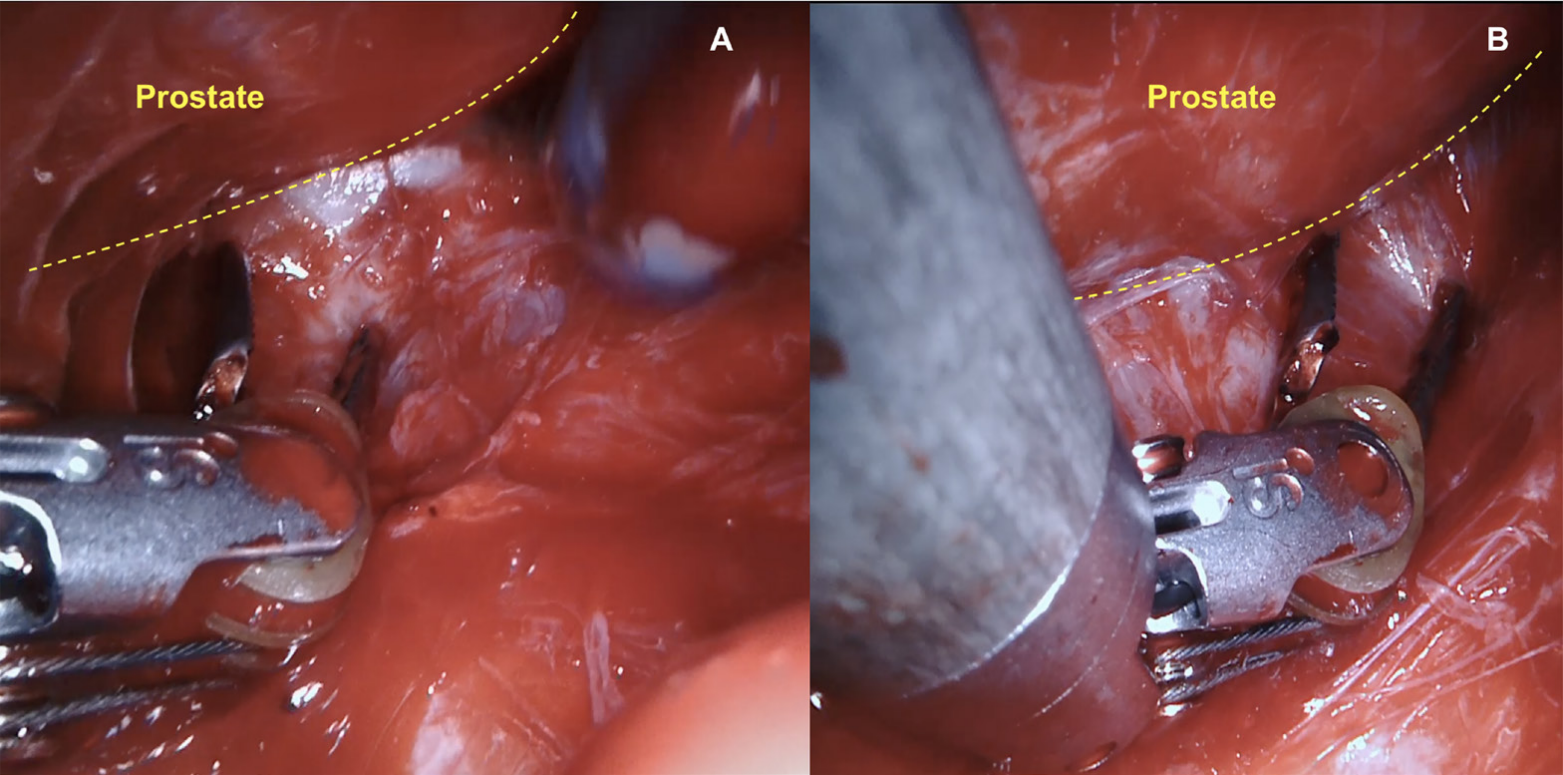
Posterior prostatic dissection from 5 to 1 o’clock on the right side (B) and from 7 to 11 o’clock on the left side (A).

Our first step to initiate the NVB preservation starts with the posterior dissection of the prostate between the Denonvilliers layers. Performing a wide posterior dissection between this avascular tissue facilitates identifying the lateral plane of dissection before controlling the arterial pedicles of the prostate. Figure-1 illustrates the posterior medial prostatic artery, one of the landmarks used to guide the different Grades of NVB preservation (15). During this step, we avoid using cautery energy while minimizing the traction of the neural bundles. Once we identify the arterial landmarks, the dissection is performed from 5 to 1 o’clock on the right side and from 7 to 11 o’clock on the left side (Figure-2). In cases that the arterial landmarks are not identifiable, we cautiously dissect the posterior plane at the artery topography avoiding entering the prostatic capsule.

**Figure 3:**
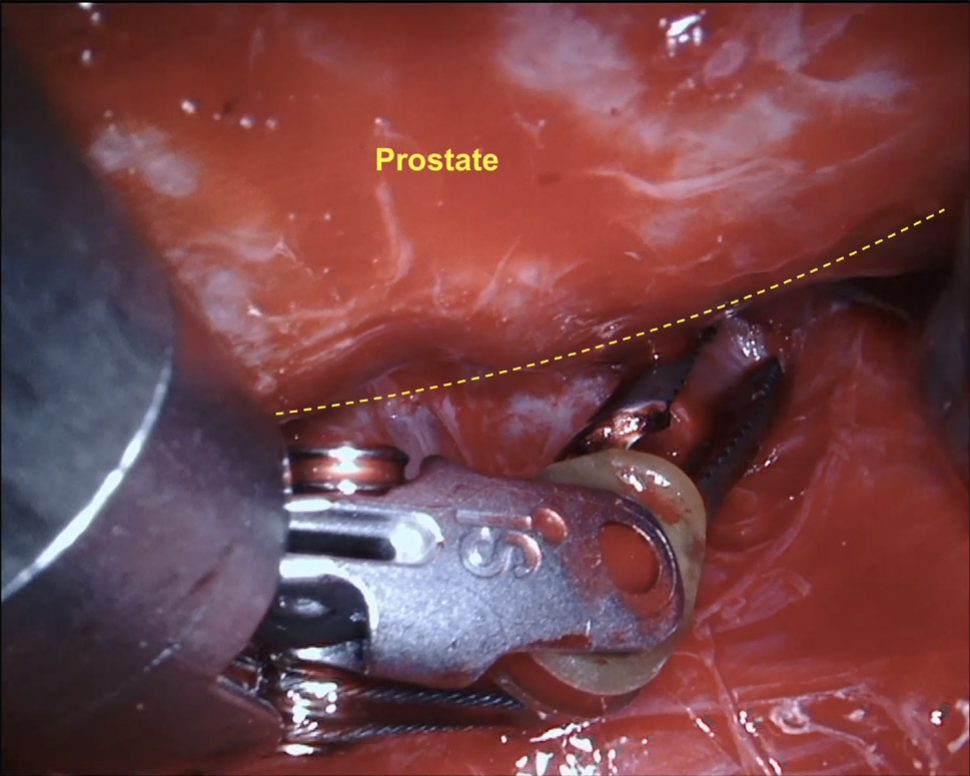
Scope facing the posterior aspect of the prostate (30-degrees up), achieving the optimal anatomical visualization necessary to release the NVB on both sides.

**Figure 4:**
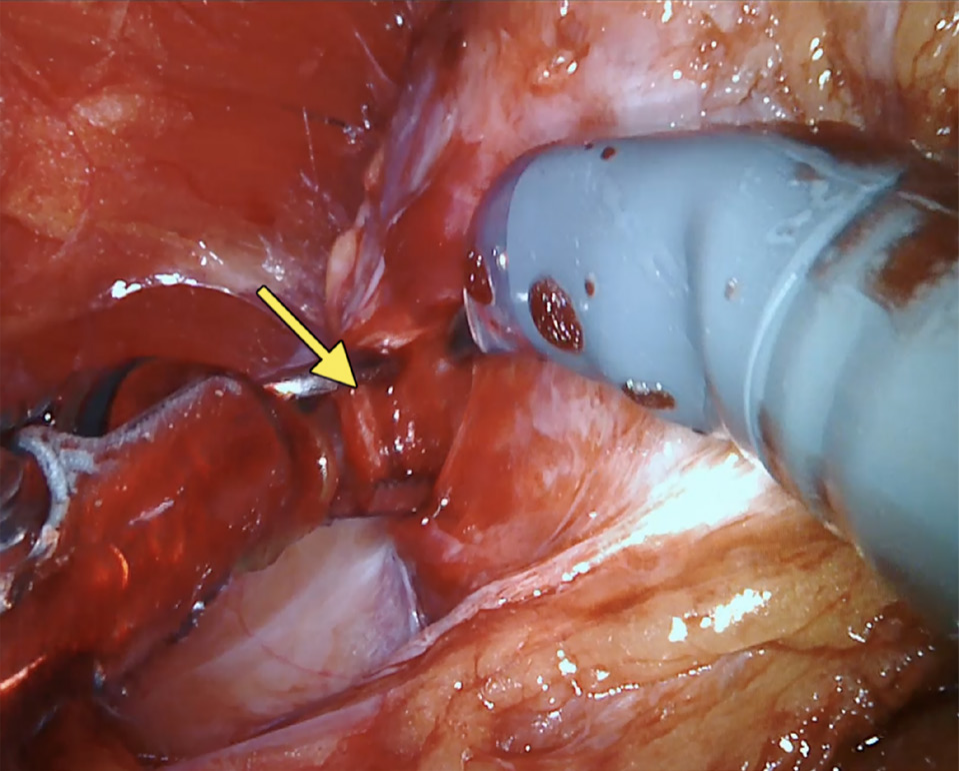
Left endopelvic fascia opening at the lateral aspect of the prostate, searching for the anteromedial prostatic artery.

**Figure 5:**
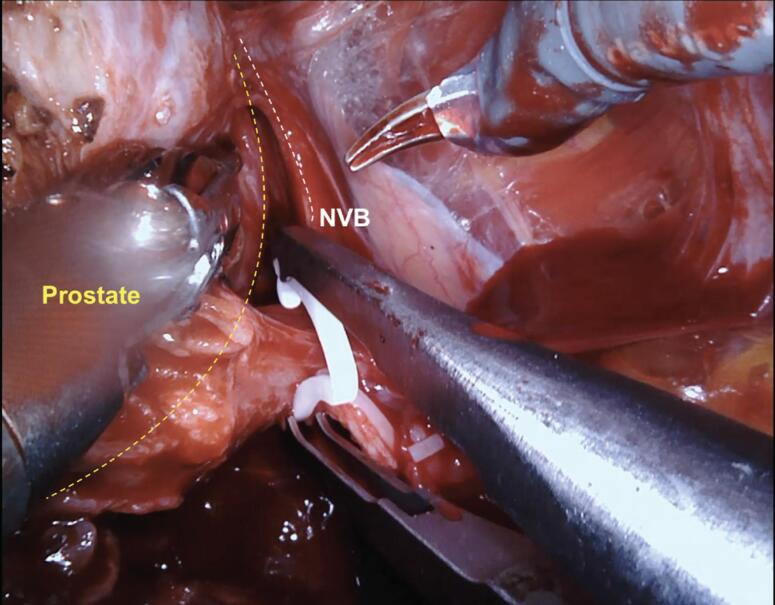
Connection between the anterior and posterior planes to isolate and ligate the prostatic pedicle with hem-o-lok clips.

**Figure 6:**
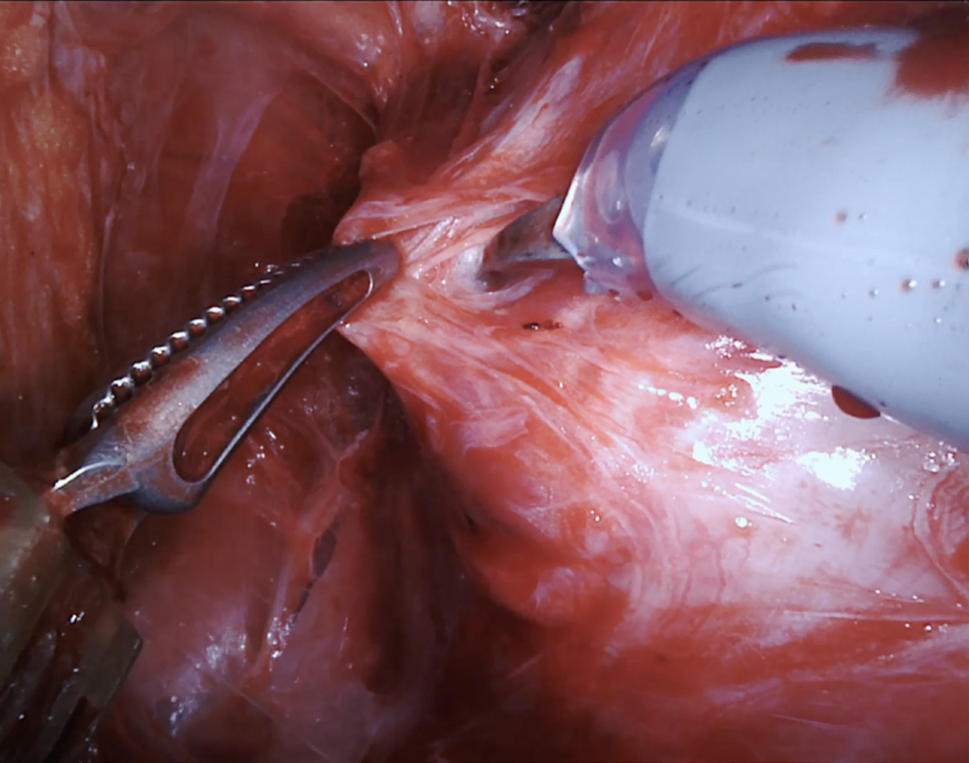
Anterior lateral prostatic dissection at the artery topography until communicating the lateral and posterior planes.

In our NS technique, the Xi scope (30-degrees) plays a crucial role during the posterior dissection between the DNV fascia. Using the toggle command, the scope faces the posterior aspect of the prostate (30-degrees up), achieving the optimal anatomical visualization necessary to release the NVB on both sides (Figure-3). Finally, we open the endopelvic fascia at the lateral aspect of the prostate, searching for the anteromedial prostatic artery, which guides the Grades of NVB dissection (Figure-4). When accessing this plane, it is usually possible to visualize the hematoma at the prostate base due to the previous posterior dissection. In sequence, we connect the anterior and posterior planes to isolate and ligate the prostatic pedicles with hem-o-lok clips (Figure-5). If the arterial landmark is not identifiable, we cautiously peel the anteromedial portion of the prostate at the artery topography until communicating with the posterior plane (Figure-6).

### Full nerve-sparing considerations

**Figure 7:**
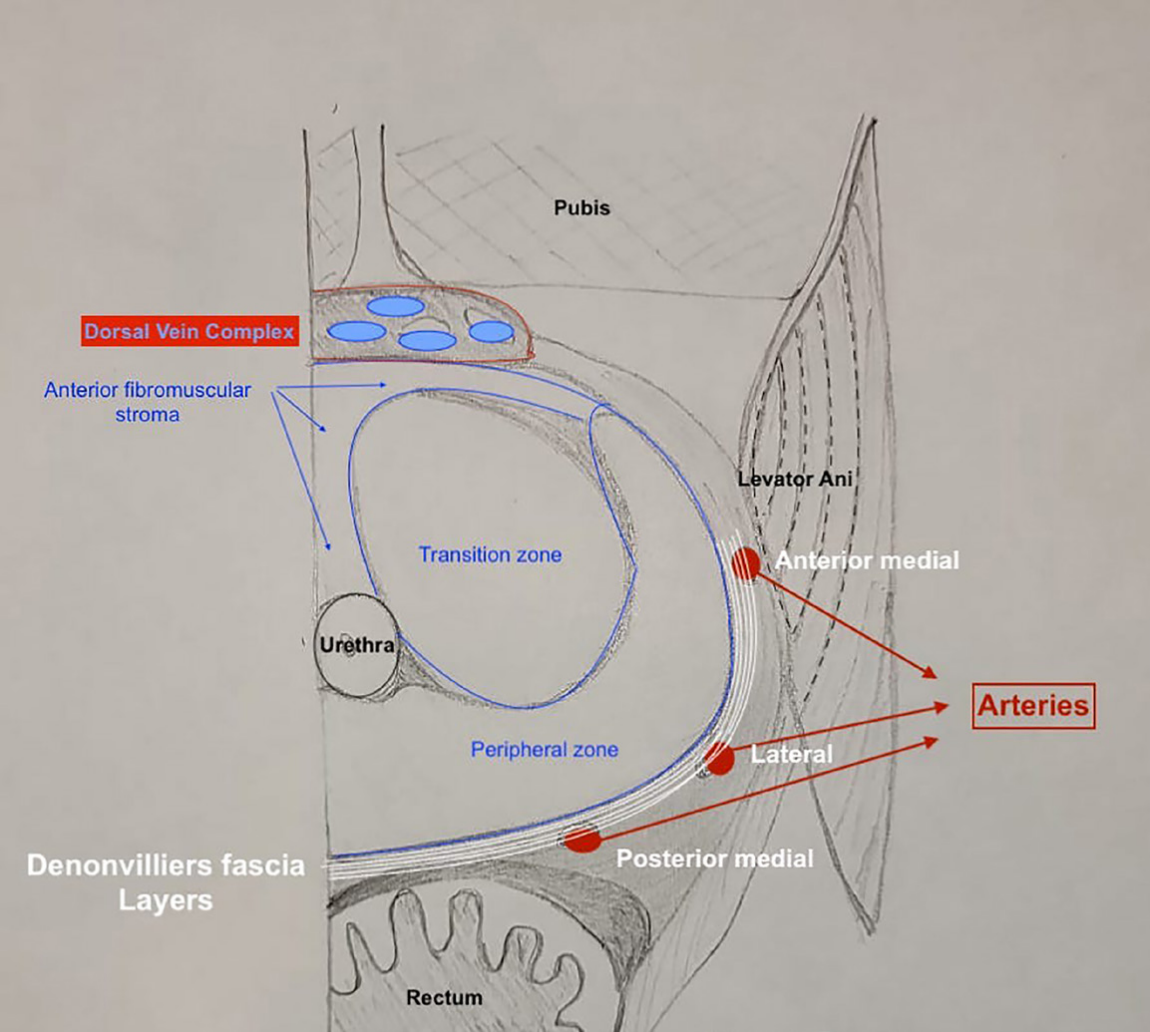
Prostate anatomy describing the arterial landmarks used to guide the nerve-sparing on the right side.

**Figure 8:**
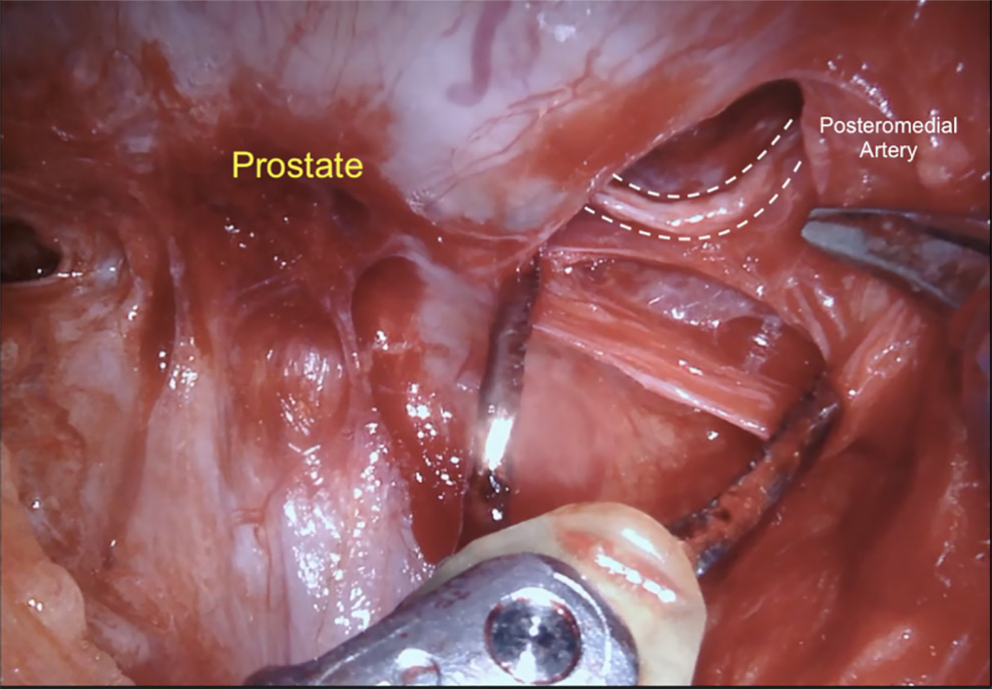
Posterior view under the prostate illustrating the posterior medial artery.

In patients undergoing full nerve-sparing, we use the landmark arteries (Figure-7), especially the posterior medial (visualized during the posterior dissection) (Figure-8) and anterior medial (visualized during the lateral dissection) (Figure-4), to guide our dissection plane. By dissecting the medial portion of these arteries closer to the prostate, we can achieve 100% NVB preservation and the best outcomes for postoperative potency.

### Partial nerve-sparing considerations

**Figure 9:**
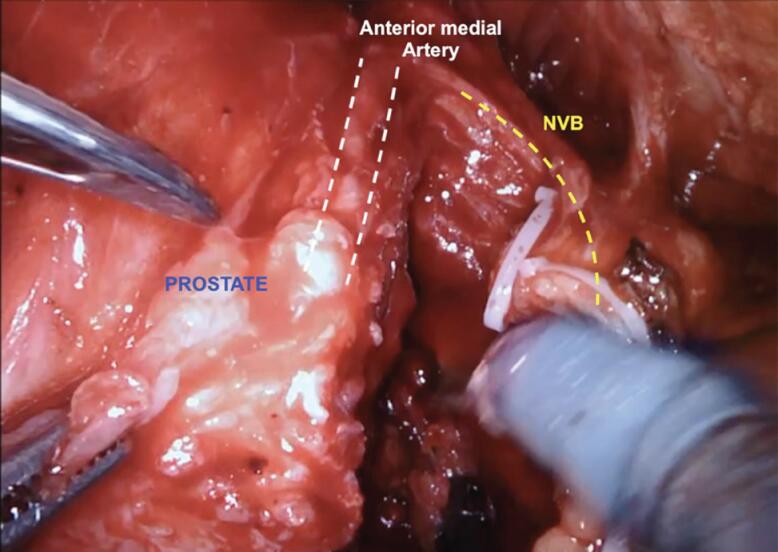
Right side of the prostate describing the anterior medial artery attached to the prostate after a partial nerve-sparing.

In patients with aggressive tumors or MRI imaging suggesting NVB invasion, we usually perform a wider dissection but still achieving a degree of neural preservation. In such cases, the dissection is guided using the lateral plane of the anterior medial and posterior medial arteries. Therefore, the arteries can be visualized attached to the prostate in the pathology analysis (Figure-9).

## CONCLUSIONS

After performing more than 15,000 cases, we believe that the NS-RARP learning curve and surgical technique are continuously evolving because the rates of postoperative functional and oncological outcomes are still inferior to 100%. Evaluating the results of our previous techniques is a crucial factor in identifying surgical steps that can be modified and improved. In addition, it is vital to know the prostate anatomy and physiology to respect the planes with careful dissection. We also consider that basic concepts, such as minimizing the amount of traction used on dissection, avoiding excessive cautery (energy) during hemostasis, and neural preservation based on anatomical landmarks (arteries and planes of dissection), should be common to all Nerve-sparing techniques.
